# First person – Ivo de Vos

**DOI:** 10.1242/bio.057935

**Published:** 2020-12-29

## Abstract

First Person is a series of interviews with the first authors of a selection of papers published in Biology Open, helping early-career researchers promote themselves alongside their papers. Ivo de Vos is first author on ‘The novel zebrafish model pretzel demonstrates a central role for SH3PXD2B in defective collagen remodelling and fibrosis in Frank-Ter Haar syndrome’, published in BiO. Ivo conducted the research described in this article while a Research Fellow in Professor Maurice van Steensel's lab at the Skin Research Institute of Singapore (SRIS), Agency for Science, Technology and Research (A*STAR), Singapore. He is now a Postgraduate House Officer in Clinical Genetics, currently working in patient care in the Department of Genetics, at the University Medical Center Groningen (UMCG), The Netherlands, investigating pathophysiological mechanisms underlying common skin conditions by studying rare genetic skin disorders, ultimately improving patient care.


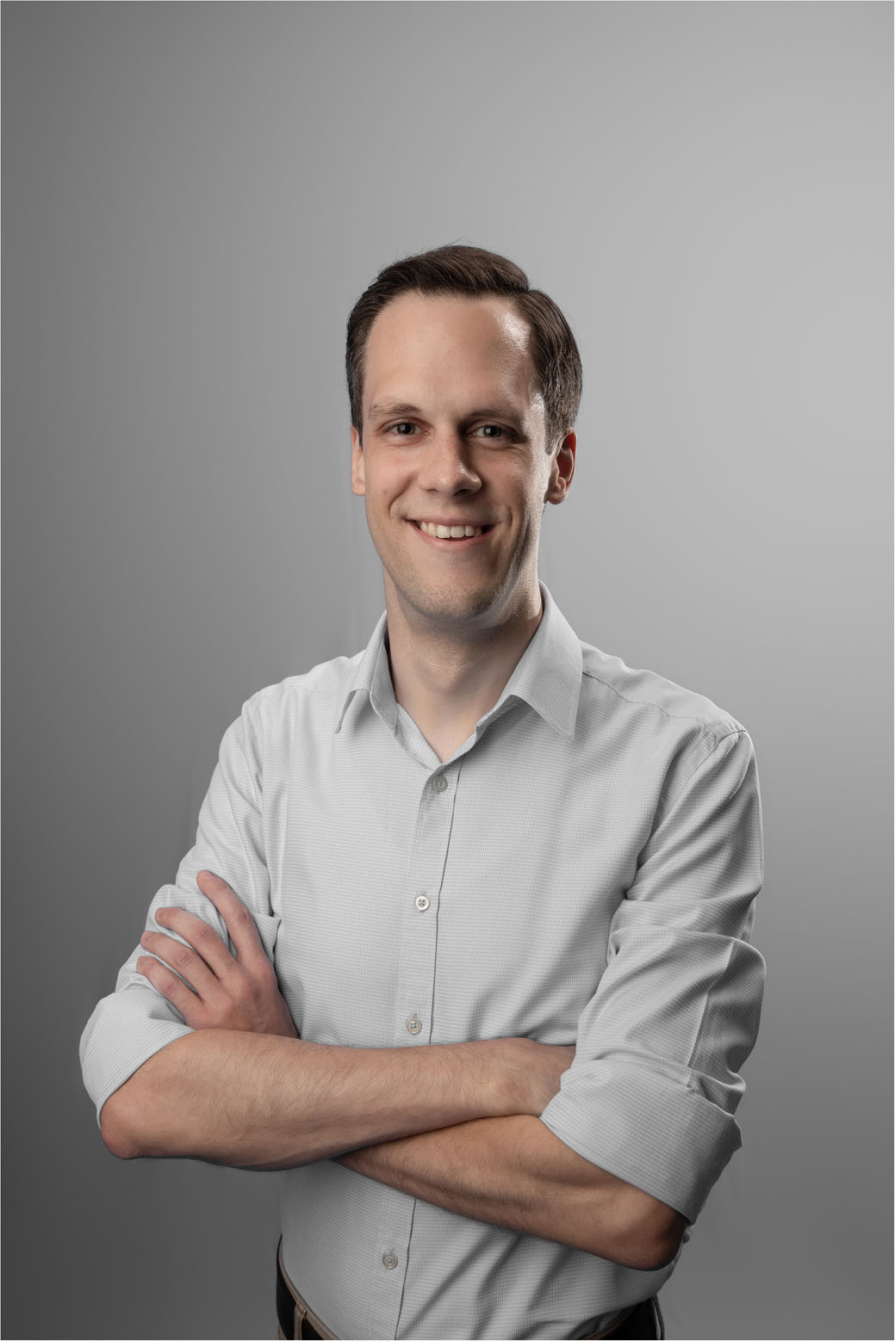


**Ivo de Vos**

**What is your scientific background and the general focus of your lab?**

My academic education started with the bachelor programme in Molecular Life Sciences at the Maastricht University. Although I was fascinated by the idea of explaining disease by disturbed molecular and cellular processes, I preferred a stronger link to clinical practice. After graduating I therefore enrolled in Medicine at the same university. During my clinical clerkships I was particularly intrigued by genetic skin disorders. The fact that their directly visible clinical presentation could be explained by the absence or malfunction of a single protein fascinated me and made me decide to pursue a PhD in molecular dermatology. The focus of my previous lab, the Acne and Sebaceous Gland Programme led by Professor Maurice van Steensel, is sebaceous gland homeostasis and acne pathogenesis. Within this lab, our subgroup aimed to gain insights into this common skin disorder by generating novel *in vitro* and *in vivo* models of rare genetic disorders associated with acne. As part of this endeavor, we generated the *pretzel* zebrafish model.

**How would you explain the main findings of your paper to non-scientific family and friends?**

The Frank-Ter Haar syndrome is a rare heritable disorder. People with this syndrome develop bone abnormalities and a gradual increase of connective tissue that limits the motion of their joints and thickens their skin. How or why exactly these abnormal changes occur, is not known. To answer these outstanding questions, we aimed to generate a zebrafish model for this human disorder. We hereto altered the genetic code of the zebrafish, similar to the genetic changes found in people with Frank-Ter Haar syndrome. In this paper, we show that we managed to successfully generate a zebrafish that mimics important features of the Frank-Ter Haar syndrome. Although the fish had relatively mild skeletal abnormalities, it developed massive build-up of connective tissue in its muscles and skin, making its fins stiffer and physically folding the fish's body axis. As such, we named this model *pretzel*, after the folded pastry.

“… we managed to successfully generate a zebrafish that mimics important features of the Frank-Ter Haar syndrome.”

**What are the potential implications of these results for your field of research?**

Our zebrafish model highlights the importance of SH3PXD2B for normal collagen homeostasis. Although the *pretzel* zebrafish reflects the soft tissue fibrosis observed in Frank-Ter Haar syndrome, the exact underlying mechanism remains unknown. As such, additional studies are needed. I believe the *pretzel* zebrafish would be a great starting point for such studies. In addition, this vertebrate model could be used to develop new drug therapies against the fibrosis observed in Frank-Ter Haar syndrome.

“Our zebrafish model highlights the importance of SH3PXD2B for normal collagen homeostasis.”

**What has surprised you the most while conducting your research?**

At first, we were under the impression that our *sh3pxd2b*^Δ*/*Δ^-mutant zebrafish only had a rather mild phenotype. On closer inspection, we observed a small white patch at the base of their dorsal fin. This seemingly minor discoloration subsequently led to the discovery of increasing dermal and musculoskeletal fibrosis, the latter thought to be responsible for the grotesque deformities characterizing the older adult mutants. This stresses the importance of a detailed phenotypical characterization of any novel mutant animal model and the value of re-evaluating animals at multiple stages throughout their life.

**Figure:**
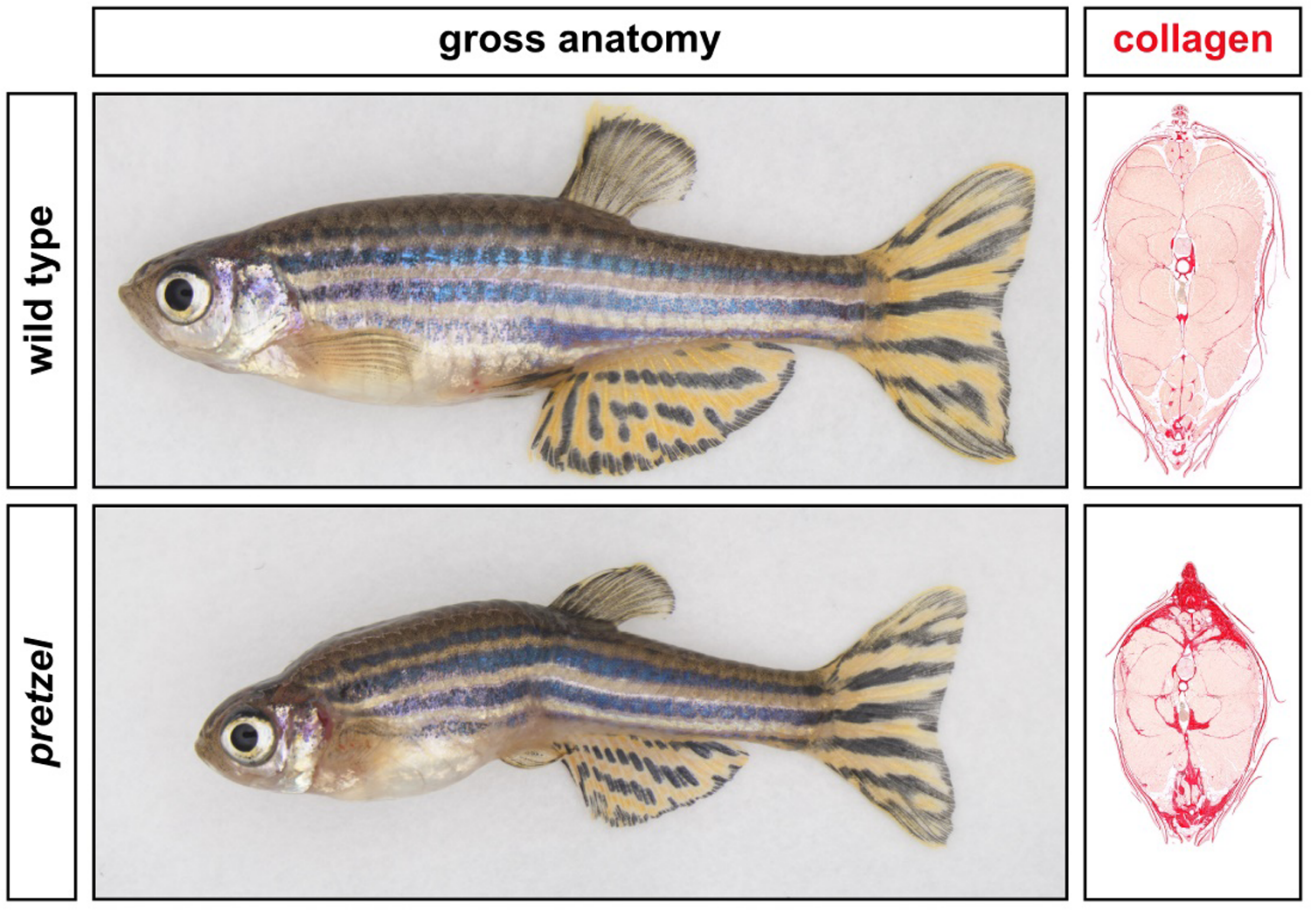
**By the age of 11 months, the *sh3pxd2b*^Δ/Δ^-mutant zebrafish *pretzel* develops extensive fibrosis, resulting in stiffer fins and a folded body axis.**

**What, in your opinion, are some of the greatest achievements in your field and how has this influenced your research?**

One of the greatest achievements in my field in recent years would be the expansion of the genome editing toolkit with the CRISPR/Cas technique. This has greatly advanced the targeted editing of genes and enabled scientists to create many novel animal models, including our currently presented zebrafish model.

**What's next for you?**

After working in science for nearly 6 years in Singapore, I have recently returned to the Netherlands to pick up clinical practice. Having a passion for both scientific research and patient care, it is my ultimate goal to combine both in the field of genetic skin disorders. I think it would be most valuable and satisfactory to encounter patients that can inspire novel lines of research, leading to results that in turn can advance patient care.

## References

[BIO057935C1] de Vos I. J. H. M., Wong A. S. W., Taslim J., Ong S. L. M., Syder N. C., Goggi J. L., Carney T. J. and van Steensel M. A. M. (2020). The novel zebrafish model *pretzel* demonstrates a central role for SH3PXD2B in defective collagen remodelling and fibrosis in Frank-Ter Haar syndrome. *Biology Open.* 9, bio054270 10.1242/bio.054270 33234702 PMC7790187

